# Pyloric Atresia Type II 

**Published:** 2013-07-01

**Authors:** Sushmita Bhatnagar

**Affiliations:** HOD, Pediatric Surgery, B.J.Wadia Hospital for Children, Parel, Mumbai, India.

**Keywords:** Pyloric atresia, Type 2, Neonate

## Abstract

Successful management of a neonate with type II pyloric atresia is reported and the relevant literature has been briefly reviewed.

## INTRODUCTION

Gastric outlet obstruction in a neonate can occur due to several etiologies. Of the entire spectrum of anomalies, pyloric atresia with complete obliteration of the pyloric canal (Type 2 pyloric atresia) is a very rare condition. Successful management is dependent on recognition of the entity, early and appropriate surgical intervention, optimum post-operative care and absence of associated anomalies. In the case described here, though there was delay in referral of the neonate, mortality (which could be as high as 50%) could be avoided by preventing sepsis, adequate electrolyte and acid-base correction and total parenteral nutrition in the post-operative period. 

## CASE REPORT

A 8-day-old boy weighing 1.9kg, born preterm at 32 weeks gestation as a normal delivery with a birth weight of 2.3kg with good Apgar score, was referred for non-bilious, non-projectile vomiting of curdled milk immediately after feeds. Examination revealed upper abdominal distension with visible peristalsis from left to right (Fig.1). An erect X ray of the abdomen (Fig. 2) showed a dilated stomach and no gas in the abdomen distal to the pylorus. An air contrast study was done which again showed a dilated stomach and no gas distally. An ultrasound abdomen showed gaseous distended stomach with a short segment thickening of pyloric wall obliterating the pyloric lumen. Following correction of electrolyte and acid base imbalance, with a diagnosis of pyloric obstruction, suspected pyloric atresia, the neonate was explored to find a congenital pyloric atresia with no gap between the two ends. Resection of the pylorus with anastomoses of the antrum of the stomach with the first part of the duodenum was done safeguarding the structures posterior to the first part of the duodenum. Broad spectrum antibiotics and total parenteral nutrition through a central line were the mainstay of post-operative management. The post-operative recovery was thus uneventful and the baby was discharged on 12th post-operative day on full breast feeds.

**Figure:**
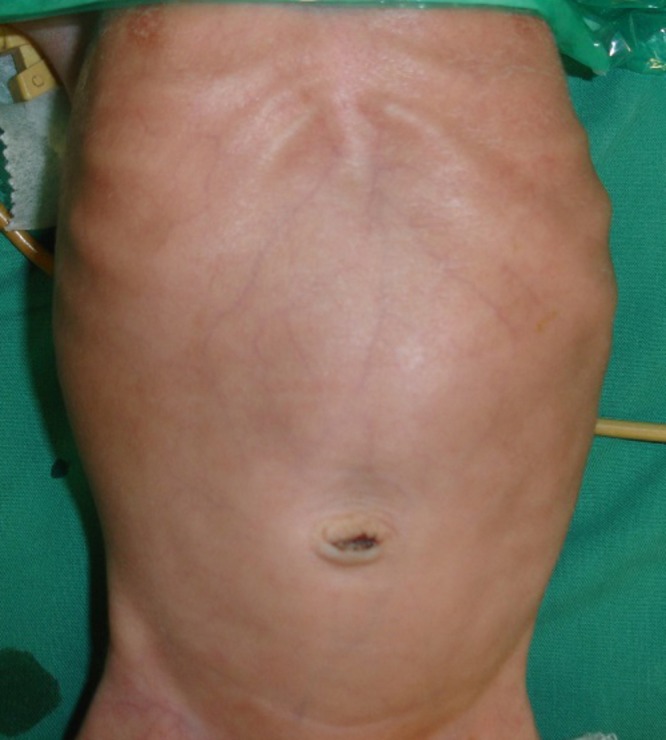
Figure 1: Upper abdominal distension due to dilated stomach with visible peristalsis.

**Figure:**
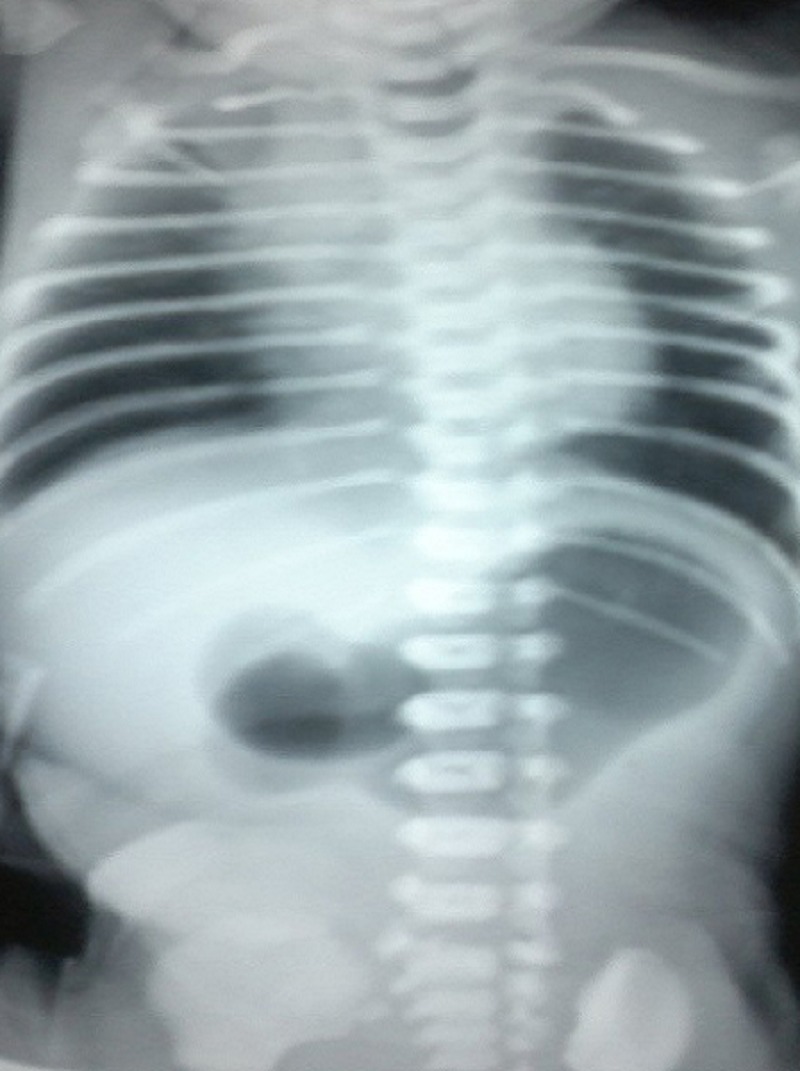
Figure 2: Dilated body and antrum of the stomach with no gas in the distal bowel loops.

## DISCUSSION

The rarity of Congenital Pyloric Atresia (CPA) i.e. 1 in 100,000 newborns, makes the clinician suspicious of other pathologies causing obstruction at the pylorus in the neonate. There are many congenital and acquired causes of gastric outlet obstruction in infants such as infantile hypertrophic pyloric stenosis, gastric volvulus, gastric antral web, congenital complete or incomplete prepyloric mucosal diaphragms [1], pyloric duplication cysts, pyloric atresia, prepyloric webs and rings [2], gastric polyps (close to the pylorus) [3], and in older children gastric and epigastric neoplasms [4] (lymphomas, gastrointestinal stromal tumors, etc.), bezoars, eosiniphillic gastritis, caustic ingestion, peptic ulcer disease, etc. [5, 6].

In newborns, apart from CPA, gastric outlet obstruction has been found to occur due to ectopic pancreas [7, 8], antral mucosal valve [9], antral diaphragm [10], prostaglandin therapy [11], gastric adenomyoma [12], and gastric duplication cyst [13]. Detailed investigations are needed for appropriate pre-operative diagnosis and identifying the exact cause of gastric outlet obstruction. 

CPA occurs in two forms – isolated CPA and associated with other anomalies including genetic. The prognosis of isolated CPA is much better than the one with associated anomalies such as epidermolysis bullosa [14-16] and hereditary multiple atresia syndrome (HMAS) [17, 18].

Three anatomical types of pyloric atresias are described [19]:

1. Type 1 – Pyloric membrane (57%)
2. Type 2 – Pyloric tissue replaced by solid tissue (34%)
3. Type 3 – Atretic pylorus with a gap between stomach and duodenum (9%)

Surgical intervention with appropriate procedure as per the type of pyloric atresia is the treatment for this condition. The mortality depends on the post-operative care of the neonates and co-existing morbidities and anomalies, which could be as high as 50% [20]. 

## Footnotes

**Source of Support:** Nil

**Conflict of Interest:** The author is editor of the journal. However, manuscript was processed by other editors and she was not involved in decision making regarding the manuscript.

